# Tobacco attributable Disability-Adjusted Life Years (DALYs) burden in Poland and Hungary: The role of women

**DOI:** 10.18332/tid/131265

**Published:** 2021-01-08

**Authors:** Paweł Koczkodaj, Joanna Didkowska

**Affiliations:** 1 Cancer Epidemiology and Primary Prevention Department, Maria Sklodowska-Curie National Research Institute of Oncology, Warsaw, Poland; 2 National Cancer Registry, Maria Sklodowska-Curie National Research Institute of Oncology, Warsaw, Poland

**Keywords:** lung cancer, tobacco, women, cancer prevention, mortality

**Dear Editor,**

**Figure 1:**
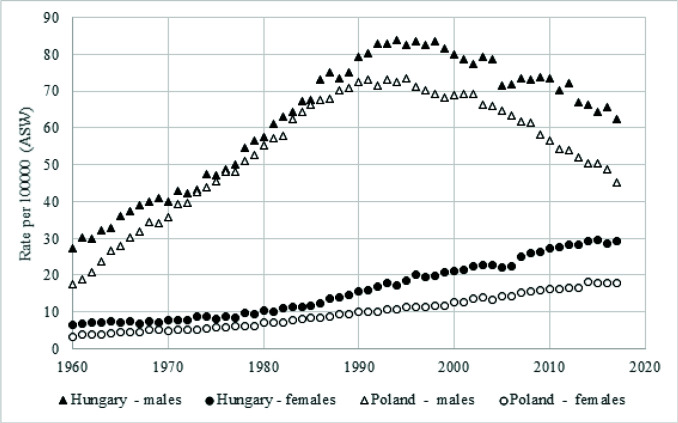
Lung cancer mortality by sex in Poland and Hungary, 1960–2017

Our letter relates to the article entitled ‘Gender differences and smoking cessation in the Japanese smoking cessation treatment program’^1^. Despite the many successes in tobacco prevention and control, smoking remains one of the biggest public health challenges – often in women populations regardless of the region of the world. This fact has been demonstrated once more by the data of the Global Burden of Disease Study^2^ published mid October 2020. Going through their analysis, we were exceptionally concerned because of the unfavorable trends in tobacco consumption in Poland and Hungary. These are two of the few European Union countries with a high DALYs burden due to tobacco use, and the highest in Central-Eastern Europe^2^. It has been investigated that to a large extent the burden is linked to lung cancer, which can be treated as an indicator disease for a great majority of other tobacco-related diseases^3^. Furthermore, looking at the World Health Organization (WHO) data on lung cancer mortality in Poland and Hungary, we notice significant differences between the male and female populations (Figure 1)^4^. While lung cancer mortality started to decrease among males since the 1990s in both countries, in the female populations a constant increase in mortality has been observed from the 1960s for both countries. The current picture of epidemiological trends is a result of smoking behaviors in the Polish and Hungarian populations in the past. Considering these data, it can be assumed that the high DALYs burden for these two countries is significantly connected to smoking behaviors of women. This phenomenon is a valuable indicator to focus on by national public health stakeholders and decision makers to protect women’s health and in order to decrease the DALYs burden in Poland and Hungary but also globally.
